# Relapsed/Refractory Non-Hodgkin Lymphoma: Engineering T-Cells to Express Chimeric Antigen Receptors (CARs), a Salvage?

**DOI:** 10.7759/cureus.16307

**Published:** 2021-07-10

**Authors:** Bushra Kanwal

**Affiliations:** 1 Internal Medicine, Brookdale University Hospital Medical Center, Brooklyn, USA; 2 Lombardi Comprehensive Cancer Center, Georgetown University Medical Center, Washington, D.C., USA

**Keywords:** non-hodgkin lymphoma, chimeric antigen receptors, immunotherapy, cd19, t cells, car t cell therapy

## Abstract

For years, patients with B-cell non-Hodgkin lymphoma (NHL) have been treated with traditional first-line therapies with a fairly acceptable outcome. However, some individuals with relapsed or resistant lymphoma do not respond to those treatments, including chemotherapy, immunotherapy, radiotherapy, and (or) autologous stem cell transplantation. Based on the acquired immunotherapy knowledge, T-cells genetically engineered with chimeric antigen receptors (CARs) seem to offer complete, enduring clinical responses to patients with refractory or relapsed lymphomas. Currently, four autologous CD19-directed CAR T-cell therapies have gained approval by the U.S. Food and Drug Administration (FDA) for the treatment of relapsed or refractory diffuse large B-cell lymphoma (DLBCL), primary mediastinal B-cell lymphoma, high-grade B-cell lymphoma, and transformed follicular lymphoma, while further CAR T-cell immunotherapies have entered the clinical trials pipeline. This review aims to summarize the efficacy and safety of the FDA-approved CAR T-cell treatments for the relapsed or refractory lymphomas.

## Introduction and background

The umbrella term B-cell lymphoma covers various malignant types defined by an irregular proliferation of the lymphoid cells at varying stages of differentiation, including Hodgkin lymphomas (HLs) and B-cell non-Hodgkin lymphomas (B-NHLs) [1]. According to the National Cancer Institute, until 2020, it was expected that approximately 77,240 new cases of non-Hodgkin lymphomas (NHLs) would have occurred, and nearly 20,000 patients would have died of the disease. This estimation was calculated based on 19.6 reported cases per 100,000 individuals annually during 2013-2017. Respectively, the death rate reached 5.4 per 100,000 individuals per year between 2014 and 2018. Both rates have been adjusted for age and sex [2]. Therefore, proper, timely management of the disease is of utmost importance. To date, conventional first-line treatment comprises rituximab in conjunction with doxorubicin, cyclophosphamide, vincristine, and prednisone (R-CHOP). Although such therapies are proved to be beneficial, the five-year survival rates do not exceed 70%, whereas, simultaneously, almost half of the patients become resistant to or experience a relapse following treatment. In these cases, the median overall survival (OS) declines drastically and does not go beyond 10 months [3]. To this extent, second-line treatment like chimeric antigen receptor (CAR) T-cells should be considered in this group of patients.

CAR T-cell treatment employs gene transfer knowledge to modify patients' T-cells to express a CAR to be directed against specific cancer cells. It is reported in the literature that for relapsed and refractory NHL, the CAR T-cells therapy has spiked the cure rate from 10% to 40% [4].

Given those encouraging outcomes, a number of clinical trials investigating the efficacy of this treatment are in progress [5]. In this review, we present the structural features and mechanism of action of CAR T-cell therapy, along with four corresponding regimens approved by the Food and Drug Administration (FDA), with a brief outline of their therapeutic and safety profile in relapsed or refractory NHL cases.

## Review

Engineering patients' immune cells for the treatment of malignancies

Immunotherapy, primarily described as a treatment that utilizes a patient's immune system to target cancer cells, has become a mainstay in tumor biology and malignancy management. One renowned advancement, the engineered CAR T-cells, was conceptualized forty years ago by Zelig Eshhar and has evolved into an emerging and promising therapeutic option in immuno-oncology thereafter [6].

Typically, T-cells destroy cancer cells; in some cases, however, T-cells space tumor cells as they might resemble normal cells of the body or they might not induce under certain circumstances immune response, leading to cancerous growth. CAR T-cells remedy this shortcoming and enhance the immune system by attaching a specific receptor facilitating T-cells to recognize and attack cancer cells. Chimeric antigen receptor is a type of engineered T-cell receptor that is able to identify a predetermined target antigen and present it to the T-cell to stimulate its cytotoxicity against the target cells. CAR T-cell identifies tumor surface antigen in an antibody-like detection model, which is not dependent on the major histocompatibility complex (MHC) [7].

CAR T-cell therapy: structural features and mechanism of action

Chimeric antigen receptor consists of four domains: 1. the extracellular domain, 2. the hinge or spacer domain, 3. the transmembrane (TM) domain, and 4. the intracellular signaling/activation domain. The extracellular domain is generally formed by the Single-chain Variable Fragment (ScFV) part of a particular antibody aiming at the target antigen. The ScFV binds to the antigen, stimulating the CAR T-cell. In turn, the activated CAR T-cell releases the relevant cytokines and other soluble mediators that may contribute to the elimination of the antigen-expressing target cells [8]. The spacer domain, on the other hand, is typically synthesized from IgG1 and affects the flexibility of the extracellular domain and function of the CAR T-cell. The transmembrane domain is obtained mainly from CD8/CD28 and modifies the expression of CAR on the T-cell membrane. The intracellular domain comprises the CD3 signaling pathway, activating the T-cell once it is bound to the target cell. Finally, co-stimulatory domains (CD28, 4-1BB, etc.), which are employed to produce the latest CAR T-cells' latest technology, can enhance the proliferation, cytokine generation, anti-tumor effectiveness, and persistence of the T-cell by delivering the secondary signaling pathway [9].

Orchestration and stages

**Figure 1:**
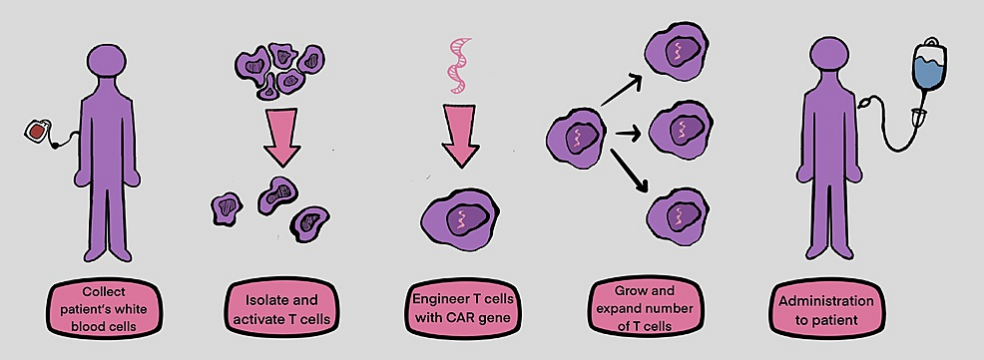
CAR T-cell therapy: orchestration and stages CAR: chimeric antigen receptor

The orchestration of engineering and delivery of CAR T-cell therapy consists of the following stages: 1. Collection, where the patient's autologous T-cells are isolated and collected via apheresis, 2. T-cell stimulation, where antibody-coated beads act as artificial dendritic cells and stimulate the isolated T-cells, 3. Transduction, where lentiviral DNA is incorporated into the activated T-cells and reprogramed into CAR T-cells, 4. Expansion, where genetically reprogrammed T-cells go through additional ex vivo expansion. When the number of CAR T-cells is sufficient, they are frozen and delivered to the healthcare facility, 5. Chemotherapy, where, prior to T-cell infusion, the patient receives a short course of preparatory chemotherapy, known as lymphodepletion, and 6. CAR T-cell infusion, where the genetically engineered T-cells are finally infused into the patient (Figure 1). Notably, through this therapy, CAR T-cells may eliminate all tumor cells and could exist in the tissues months after the infusion is complete. That might explain the reason why this treatment leads to long-lasting remission for certain types of blood cancer [10].

FDA-approved CAR T-cell therapies

In NHL, CD19 is the target antigen that lymphoma treatment is based on. Anti-CD19 CAR T-cells have doubtlessly changed the landscape of relapsed or resistant NHL management. Building on this concept, novel regimens and clinical trials have been designed using the CD19 antigen as key pattern-matching criteria to recognize and eradicate tumor cells [5]. We provide below a short review of four already FDA-approved CAR T-cell treatments.

Tisagenlecleucel (Kymriah™) 

In 2018, tisagenlecleucel, under the brand name Kymriah (Novartis Pharmaceuticals Corp., East Hanover, New Jersey), previously known as CTL019, gained approval by the FDA for adults with relapsed or refractory large B-cell lymphoma. Tisagenlecleucel is a second-generation anti-CD19 genetically modified autologous T-cell immunotherapy with a 4-1BB activating domain. A lentiviral vector is utilized for the transduction of autologous T-cells to produce Kymriah. It is suitable for patients that failed more than two lines of systemic treatment, including diffuse large B-cell lymphoma (DLBCL) not otherwise specified, DLBCL arising from follicular lymphoma, and high-grade B-cell lymphoma [11].

Approval was based upon JULIET, a single-arm, open-label, multicenter, phase II study, investigating the efficacy and safety of tisagenlecleucel in adult patients with relapsed or resistant DLBCL and DLBCL after transformation from follicular lymphoma [12]. The results of the trial showed that patients treated with tisagenlecleucel achieved overall (OR) and complete response (CR) in a rate of 53.1% and 39.5%, respectively, while complete response rates (CRR) at three months and six months of follow-up were 32% and 30%, respectively. Six months after treatment initiation, a complete response was sustained in 95% of patients, and the possibility of overall survival was calculated to 64.5%. As regards the safety profile, 86% of patients experienced grade 3 or higher toxicities, according to the University of Pennsylvania grading. Grade 3 and higher cytokine release syndrome (CRS) reported in 23% of patients without any grade 5 events. Grade 3 and higher neurotoxicity happened in 12% of patients with no grade 5 incident. Three deaths were reported within one month of infusion because of disease progression [13]. In May 2018, the FDA authorized a priority review of supplementary Biological License Application (sBLA) for Kymriah for the management of relapsed or refractory DLBCL [14].

Axicabtagene ciloleucel (Yescarta™)

On October 18, 2017, the FDA approved axicabtagene ciloleucel, marketed as Yescarta (Kite Pharma Inc., Los Angeles, California), is a CD19-directed genetically modified autologous CAR T-cell therapy designated for the management of adults with relapsed/refractory large B-cell lymphoma after two or more lines of systemic therapy, including DLBCL not otherwise specified, primary mediastinal large B-cell lymphoma, high-grade B-cell lymphoma, and DLBCL arising from follicular lymphoma [15].

ZUMA-1, a single-arm, open-label trial of axicabtagene ciloleucel yielded excellent and durable results in efficacy in patients with refractory large B-cell lymphoma. Phase I outcomes in seven patients with DLBCL who achieved an OR rate of 71% with four out of seven (57%) obtaining a CR resulted in the pivotal, phase II of the study [16]. In the multicenter, phase II trial, 111 patients with DLBCL, primary mediastinal B-cell lymphoma, or transformed follicular lymphoma with the resistant disease despite receiving the recommended first-line treatment were recruited. Of those, axicabtagene ciloleucel was successfully engineered for almost all of them (110/111; 99%) and infused to 91% (101/111). The objective response rate (RR) was 82%, and the CRR was 54%. At the 15.4-month follow-up, 42% of the patients maintained the response, while an almost equal number of cases (40%) continued to report a complete response. The overall survival rate at 1.5 years was 52%. The most frequent side effects of grade 3 or higher during the infusion were neutropenia (78%), anemia (43%), and thrombocytopenia (38%). Grade 3 or higher CRS and neurologic incidents arose in 13% and 28% of the patients, respectively [17].

However, the medical research of axicabtagene ciloleucel is ongoing. ZUMA-5, for example, is a phase II, multicenter, single-arm study in adults with relapsed or refractory NHL. Until March 12, 2020, 146 patients of an assessed 160 study population had received the treatment. Preliminary results showed that, among the already evaluated patients (n=104), the ORR reached 92%, whereas the CR rate was reported as high as 76% after a mean follow-up of 17.5 months (range: 1.4-31.6). Patients had one-year estimated rates of 72% for the duration of response, 74% for progression-free survival, and 93% for overall survival.

During treatment, grade 3 or higher adverse reactions in 85% of the follicular lymphoma cohort and 95% of the marginal zone lymphoma cohort (86% overall) were reported. Grade 3 or higher CRS and neurologic events occurred in 6% and 15% of the follicular lymphoma group and 9% and 41% of the marginal zone lymphoma group (7% and 19% overall) and subsided by the time of data cutoff. Regarding the mortality during the study period, overall, three patients died: one related to the treatment, where a patient developed multisystem organ failure during CRS, and two unrelated to treatment and were attributed to aortic dissection and coccidioidomycosis infection [18].

Brexucabtagene autoleucel (Tecartus™)

In July 2020, the FDA gave accelerated approval to brexucabtagene autoleucel (Tecartus, Kite, a Gilead Company), formerly known as KTE-X19. Brexucabtagene autoleucel is an anti-CD19 genetically engineered autologous T-cell immunotherapy for the management of relapsed or resistant mantle cell lymphoma. Approval was granted after the promising results of the ZUMA-2 trial [19].

ZUMA-2 is an open-label, multicenter, single-arm study examining the efficacy of KTE-C19, an autologous second-generation CD19 CAR T-cell therapy consisting of a CD28 activating domain. With the anticipated initial completion date of July 2018, 74 patients with relapsed or refractory MCL were enrolled and administered eventually to 68 [20]. Of the 60 patients evaluable for the primary effectiveness analysis and after at least a six-month follow-up for the response, the ORR was as high as 87%, with a satisfactory CR rate of 62%. The most frequent (≥10%) grade 3 or higher adverse events reported were anemia, neutropenia, leukopenia, thrombocytopenia, hyper- and hypotension, hypophosphatemia, encephalopathy, hypoxia, fever, hyponatremia, infection - pathogen unspecified, pneumonia, hypocalcemia, and lymphopenia [21].

Lisocabtagene maraleucel (Breyanzi)

In February 2021, the FDA granted approval for lisocabtagene maraleucel, formerly known as JCAR 017 and currently branded as Breyanzi (Juno Therapeutics, Inc., Seattle, Washington). Lisocabtagene maraleucel is suitable for the management of adult patients with relapsed or resistant LBCL after two or more lines of systemic therapy, including DLBCL not otherwise specified, including DLBCL arising from indolent lymphoma, high-grade B-cell lymphoma, primary mediastinal LBCL, and follicular lymphoma grade 3B [22].

Approval was gained after the efficacy and safety report of the TRANSCEND NHL001 trial, an open-label, multicenter, phase I study in patients with relapsed or refractory DLBCL or MCL. According to the study, of the 192 eligible for evaluation patients, the overall RR per independent review committee assessment was calculated at 73%, with a CR rate of more than 50% (54%). The initial clinical results were evident one month on average after the infusion. Of the 104 participants who obtained CR, 65% remained in remission for more than six months, with an equivalent percentage (62%) reporting no recurrence for more than nine months. CRS was reported by 46% of patients (≥3 grade: 4%), and neurologic toxicity was experienced by 35% (≥3 Grade: 12%) of subjects, with fatal neurologic toxicity occurring in three patients. Other Grade 3 or higher severe events included some types of infection (19%) and persistent cytopenia (31%) [23].

## Conclusions

The progress in biotechnology and medicine has fueled the development of cell-based treatments for a wide range of diseases, including B-cell lymphomas. Recently, CAR T-cell regimens have emerged as a promising therapeutic option; yet, some improvements are undoubtedly required: the efficacy and persistence of adoptive cells need to be increased, and a better safety profile is essential.

Relevant clinical trials on blood cancers utilizing CAR T-cell technology have yielded exquisite results in the initial evaluation. To this extent, CAR engineering is under constant optimization while the most beneficial combination of CAR T-cell treatment with other immunotherapies or targeted therapies should be determined. Moreover, additional trials are needed to identify the ideal timing and stage to administer the engineered CAR T-cells to maximize the efficacy. Finally, the integration of genetic and molecular biomarkers may also contribute to guiding future studies and management decisions.
